# 

**Published:** 1989-08

**Authors:** 


Established 1883

### BRISTOL MEDICO-CHIRURGICAL JOURNAL

**AUGUST 1989**
**Volume 104(iii)**

Copyright © Clinical Press Ltd 1989.

**Editor**
Mr M. G. Wilson
*40 Coombe Lane, Bristol BS9 2BJ*
*Tel: 0272-682320*

**Editorial Committee**
Dr Paul Goddard (Assistant Editor)
Mr B. P. Jones (Secretary)
Dr J. L. Burton

**Correspondents**
Dr A. E. Adam (Taunton)
Mr D. C. C. Bartolo
Mr M. L. Crosfil (Penzance)
Dr J. D. Davies
Dr J. Jancar
Professor D. J. Pereira Gray (Exeter)
Dr H. G. Mather
Professor G. M. Stirratt
Mr C. Teasdale (Plymouth)
Dr J. L. G. Thomson
Dr M. J. Whitfield
Professor G. K. Wilcock

**BRISTOL MEDICO-CHIRURGICAL SOCIETY**

Mr A. John Webb (President)

Mr R. N. Baird (Secretary)
*23 Old Sneed Park, Bristol BS9 1RG*

Dr N. C. Tricks (Treasurer)
*The Mill, Wrington, Bristol*

**Published by**
**Clinical Press,**
*15 Bucklands Drive*
*Nailsea*
*Bristol*

**Typesetting by**
*Apek Typesetters*
*Avon House, Nailsea, Bristol BS19 2DJ*

**Printed by**
*Sutton Print, Paignton*

**THE BRISTOL MEDICO-CHIRURGICAL JOURNAL acknowledges with gratitude the help it receives from THE NUFFIELD HOSPITAL.**

*Scire est nescire, nisi id me alius sciret.* **Lucilius (180–102 BC)**

### THE MEDICAL JOURNAL OF THE SOUTH WEST

**NOTICE TO CONTRIBUTORS**
Contributions are invited especially from West of England authors on a variety of subjects, e.g. Original articles relating to the practice of medicine, reports on the contemporary medical scene, obituaries, historical accounts, recollections of colleagues and events worthy to be remembered and liable to be forgotten. An article of 2,000 words with 2 illustrations will occupy 2 pages and this is a suggested, though by no means an obligatory length. Articles are preferred in the form proposed by the International Committee of Medical Editors (Vancouver System)—pamphlet obtainable from Editor of BMJ., BMA. House, Tavistock Square, London, WC1H 9JR.

### Editorial

**THE ROYAL COLLEGE OF GENERAL PRACTITIONERS**

**Severn Faculty**

**The 33rd GALE MEMORIAL LECTURE**

"STANDARDS IN GENERAL PRACTICE"
*to be given by*
Dr. Donald Irvine, CBE, MD, FRCGP.

formerly Chairman of Council RCGP, and currently member of GMC.
on Saturday, 14th October, 1989 at 7.30 p.m. for 8.00 p.m.
at Tewkesbury Hall Hotel.
to be followed by

**THE ANNUAL FACULTY DINNER**

